# Editorial: Integrating machine learning with physics-based modeling of physiological systems

**DOI:** 10.3389/fphys.2025.1562750

**Published:** 2025-02-05

**Authors:** Jae H. Lee, Hao Gao, Michael Döllinger

**Affiliations:** ^1^ Division of Quantitative Methods and Modeling, Office of Research and Standards, Office of Generic Drugs, Center for Drug Evaluation and Research, US Food and Drug Administration, Silver Spring, MD, United States; ^2^ The School of Mathematics and Statistics, University of Glasgow, Glasgow, United Kingdom; ^3^ Division of Phoniatrics and Pediatric Audiology, Department of Otorhinolaryngology Head and Neck Surgery, University Hospital Erlangen, Friedrich-Alexander-Universität Erlangen-Nürnberg (FAU), Erlangen, Germany

**Keywords:** physics-based modeling, modeling and simulation (CM&S), computational medicine and biology, artificial intelligence, machine learning

## Introduction

Physics-based modeling has long been instrumental in advancing our understanding of physiological systems, including cardiovascular, neurovascular, pulmonary, and gastrointestinal systems. These models, rooted in mathematical and computational frameworks, have provided detailed insights into the underlying mechanisms of complex biological phenomena. Complementing this, machine learning (ML) has emerged as a transformative tool in computational research. By integrating these two approaches, researchers aim to overcome persistent challenges in physiological modeling and advance both scientific understanding and clinical applications.

The integration of machine learning with physics-based modeling leverages their complementary strengths: data-driven insights from ML and mechanistic understanding from physics-based models. Physics-based approaches often face bottlenecks, such as computational expense, limited grid resolution, and challenges in handling large datasets. Machine learning has shown great promise in alleviating these limitations by enhancing computational efficiency, automating data processing, and improving parameter estimation. This Research Topic explores the intersection of these methodologies, showcasing innovative applications and developments in this interdisciplinary field.

*“Intracranial Pressure-Flow Relationships in Traumatic Brain Injury Patients Expose Gaps in the Tenets of Models and Pressure-Oriented Management*”. In their study, Stroh et al. investigate intracranial pressure-flow relationships in patients with traumatic brain injury (TBI), critically evaluating the assumptions underlying current management protocols. By integrating physics-based hemodynamic models with multimodality monitoring data, the authors identify novel dynamical regimes, such as zero and negative pressure-flow relationships, where standard assumptions fail. While the study primarily focuses on validating these regimes and refining mechanistic models, it highlights the potential of data-driven approaches, including machine learning, for future personalization of TBI care. Their findings challenge traditional frameworks, providing a foundation for improving critical care through a deeper understanding of intracranial hemodynamics.

*“Pole Balancing on the Fingertip: Model-Motivated Machine Learning Forecasting of Falls”*. Depnath et al. investigate the dynamics of human pole balancing on a fingertip by combining a physics-based inverted pendulum-cart model with machine learning techniques. Using a long short-term memory (LSTM) network, the study predicts falls based on both synthetic data generated from the mathematical model and experimental human balancing data. The innovative use of transfer learning—pre-training on synthetic data and fine-tuning with real-world data—enables the model to forecast 60%–70% of falls up to 2.35 s in advance, demonstrating a notable improvement in performance. By addressing data scarcity and bridging synthetic and real-world data, the authors demonstrate how machine learning can tackle challenges in chaotic physiological systems, providing a framework applicable to domains like biomechanics and neuroscience.

*“Using Dropout Based Active Learning and Surrogate Models in the Inverse Viscoelastic Parameter Identification of Human Brain Tissue”*. Inverse parameter identification in biomechanics often requires computationally intensive finite element (FE) simulations. Hinrichsen et al. address this challenge by combining a generalized Maxwell viscoelastic FE model with a recurrent neural network (RNN) surrogate model. The surrogate approximates the output of FE simulations, significantly reducing computational costs while maintaining high fidelity. Dropout-based active learning further enhances efficiency by selecting the most informative training data. The surrogate model is applied to identify material parameters for human brain tissue, showcasing how machine learning can accelerate and streamline complex computational tasks. This study demonstrates the potential of ML in reducing computational costs while maintaining high fidelity in physics-based modeling.

*“Neural Network-Based Estimation of Biomechanical Vocal Fold Parameters”*. The biomechanics of vocal fold vibrations play a crucial role in voice physiology, yet accessing the underlying biomechanical parameters remains challenging. Donhauser et al. present a convolutional recurrent neural network (CRNN) trained on synthetic data from a six-mass model (6 MM) to estimate key biomechanical parameters, such as subglottal pressure, stiffness, and mass, from high-speed video (HSV) data. This approach replaces computationally expensive optimization with a neural network surrogate, achieving significant efficiency gains. Their work advances the field of vocal fold modeling, offering a fast and accurate method for analyzing laryngeal kinematics and improving diagnostics for voice disorders.

*“MRI-MECH: Mechanics-Informed MRI to Estimate Esophageal Health”*. In the final contribution, Halder et al. introduce MRI-MECH, a mechanics-informed neural network framework for assessing esophageal health non-invasively using dynamic MRI data. By integrating computational modeling with dynamic imaging, this approach estimates critical health metrics such as wall stiffness and active relaxation without invasive procedures. Physics-informed neural network enhances the framework’s ability to overcome limitations in traditional imaging, such as poor resolution or missing data, while ensuring that its predictions adhere to fundamental physical principles, including mass and momentum conservation equations. It demonstrates the potential of combining machine learning with physics-based modeling to improve gastrointestinal diagnostics, setting the stage for broader applications in non-invasive medical imaging and physiological analysis.

## Conclusion

The five articles in this Research Topic underscore the impact of integrating machine learning with physics-based modeling across various physiological domains. They introduce innovative solutions that merge the predictive power of ML with the mechanistic insights of physics-based models.

The integration of machine learning with physics-based modeling represents a paradigm shift in computational physiology. By bridging the gap between data-driven methods and mechanistic understanding, these approaches offer transformative potential for both research and clinical practice. Future directions may include developing hybrid models that seamlessly combine ML and physics-based components, as well as standardizing methodologies and datasets for broader applicability.

As computational power continues to grow, the accessibility of these integrated approaches will expand, enabling their application to even more complex and multiscale systems, such as molecular dynamics and organ-level systems. The studies presented here exemplify how interdisciplinary collaborations can enhance our ability to model, understand, and treat physiological systems.

In conclusion, the articles in this Research Topic represent cutting-edge developments in integrating machine learning with physics-based modeling. By addressing computational inefficiencies and enhancing predictive accuracy, they not only advance the state of the art in computational physiology but also inspire new possibilities for tackling some of the most pressing challenges in biomedical research and healthcare.

